# *Moringa oleifera* alcoholic extract protected stomach from bisphenol A–induced gastric ulcer in rats via its anti-oxidant and anti-inflammatory activities

**DOI:** 10.1007/s11356-022-20543-0

**Published:** 2022-05-12

**Authors:** Reda Abo Elfath Ahmed Abo-Elsoud, Seham Ahmed Mohamed Abdelaziz, Mabrouk Attia Abd Eldaim, Suzan Moustafa Hazzaa

**Affiliations:** 1 Department of Medical Physiology, Faculty of Medicine, Menoufia University, Shebeen El-Kom, Egypt; 2 Department of Histology, Faculty of Medicine, Menoufia University, Shebeen El-Kom, Egypt; 3 Department of Biochemistry and Chemistry of Nutrition, Faculty of Veterinary Medicine, Menoufia University, Shebeen El-Kom, Egypt

**Keywords:** Bisphenol A, *Moringa oleifera*, Gastric ulcer, Prostaglandins, Anti-inflammatory

## Abstract

This study evaluated the protective potentials of *Moringa oleifera* leaf alcoholic extract (MOLE) against bisphenol A (BPA)-induced stomach ulceration and inflammation in rats. Control rats received olive oil. Second group administered MOLE (200 mg/kg bwt) by oral gavage. Third group was given BPA (50 mg/ kg bwt) for 4 weeks. Fourth group administrated BPA and MOLE simultaneously. Fifth group was given MOLE for 4 weeks then administered BPA and MOLE for another 4 weeks. Bisphenol A induced gastric ulceration and decreased the volume of gastric juice, prostaglandin E2 (PGE2), reduced glutathione (GSH) and interleukin 10 (IL-10) contents, superoxide dismutase (SOD) activity, and proliferating cell nuclear antigen (PCNA) protein in stomach tissues, while increased the titratable acidity, malondialdehyde (MDA), tumor necrosis factor alpha (TNF-α) and interleukin 6 (IL-6) contents, and caspase-3 and NF‑κB proteins in stomach tissue. However, MOLE ameliorated BPA-induced gastric ulceration and significantly increased the volume of gastric juice, PGE2, GSH and IL-10 contents, SOD activity, and PCNA protein while significantly decreased titratable acidity, MDA, TNF-α and IL-6 contents, and of NF‑κB and caspase-3 proteins in gastric tissue. This study indicated that MOLE protected stomach against BPA-induced gastric injury via its anti-oxidant, anti-apoptotic, and anti-inflammatory activities.

## Introduction

Bisphenol A (BPA) is a toxic chemical used in manufacturing of a wide variety of products including, plastic bottles, toys, water bottles, fax paper, and canned food containers. The extensive use of this substance exposes human and animals to its toxic effects (Groff 2010) that disturb endocrine and immune systems through activating many immune pathways involved in autoimmune diseases (Kharrazian 2014). Bisphenol A has a structural similarity to estrogen; thus, it exhibits high-affinity binding to estrogen receptors affecting the reproductive system, as well as the metabolism (Szymanska et al. 2018). Since BPA can leak into food from plastic containers, humans are exposed to this substance mainly through the digestive tract. It is absorbed by the gastrointestinal tract and distributed throughout the body via the bloodstream (Almeida et al. 2018). Moreover, it has a neurodegenerative effect on the enteric nervous system (ENS) in the wall of the gastrointestinal tract affecting the stomach and intestine by decreasing the cholinergic neurons in all parts of the gastric wall (Makowska and Gonkowski 2020). BPA exerts its toxic effects through induction of oxidative stress via increasing the lipid peroxidation, inhibition of the activities of the anti-oxidant enzymes, and reducing gene expression of the anti-oxidant enzymes leading to mitochondrial dysfunction (Kaur et al. 2014; Olukole et al. 2019; Shirani et al. 2019; Hassan et al. 2012). In addition, chronic exposure of rats to BPA lead to pulmonary inflammatory diseases through increasing malondialdehyde (MDA) and IL-18 and reducing superoxide dismutase (SOD) levels in lung tissue (Abedelhaffez et al. 2017).

*Moringa oleifera* is a fast-growing tree, native to India, and was known as the miracle tree because of the nutritive values of its parts. Moreover, it has been used in medicine as a traditional herbal treatment throughout the world (Luqman et al. 2012) and micronutrients as it is rich in β-carotene, vitamins C, K, E, D, B1, B2, B3, B6, and B12, calcium, potassium, iron, isothiocyanates, and polyphenols (Bennett et al. 2003, Ufele et al. 2013). In addition, *Moringa oleifera* is rich in many anti-oxidants such as ellagic acid, apigenin, quercetin, and kaempferol (Mousa et al. 2019). Thus, it has hepatoprotective, and anti-oxidant effects in rats (Abd Eldaim et al. 2017; El Mahdy et al. 2020). To the best of our knowledge previous studies concerning the toxic effect of BPA on gastric mucosa and the protective effect of *Moringa oleifera* against gastric ulcer were rare. Thus, this study evaluated the protective potentials of *Moringa oleifera* leaf alcoholic extract against bisphenol A-induced stomach ulceration and inflammation in rats.

## Materials and methods

### Preparation of *Moringa oleifera* ethanolic extract

*Moringa oleifera* ethanolic extract was prepared and its phytochemical constituents were analyzed as described in our previous study (Mousa et al. 2019).

### Animals

Forty male Wister albino rats weighing 200–250 g were used in this study. Rats were kept on standard laboratory chow and water ad libitum, housed in well-ventilated cages (70 × 70 × 60 cm), 5 rats/cage under normal light/dark cycle and room temperature 24–25 °C. Rats were reared and treated in accordance with the experimental protocol that was approved by the local ethical committee of the Faculty of Medicine, Menoufia University with approval code 314/019 following the guide for the Care and Use of Laboratory Animals (eighth edition, National Academies Press) (Albus 2012).

### Experimental design

The animals were assigned into five equal groups, 8 rats each.

Control group: rats received 1 ml of olive oil daily by oral gavage for 4 weeks.

*Moringa oleifera* group (MOLE): rats received *Moringa oleifera* leaf alcoholic extract at a dose of 200 mg/kg bwt and olive oil for 4 weeks (El Mahdy et al. 2020).

Bisphenol group (BPA): rats received bisphenol A (Sigma Chemical Company, USA) in (1 ml) olive oil daily by oral gavage for 4 weeks (Mahdavinia et al. 2019).

*Moringa oleifera* co treated (MOLE co-treated) group: rats received both *Moringa oleifera* extract as the 2nd group and bisphenol as the third group simultaneously for 4 weeks.

**Fig. 1:**
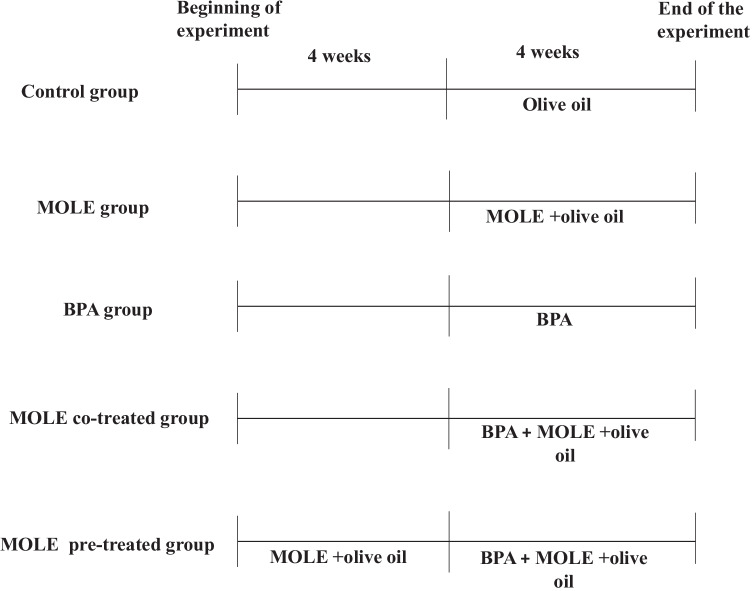
Diagram demonstrating our experimental design

*Moringa oleifera* pretreated (MOLE pre-treated) group: rats received *Moringa oleifera* extract as the 2nd group for 4 weeks then BPA and *Moringa oleifera* extract as the 4th group for another 4 weeks. Figure 1 represented the experimental design.

### Biochemical analysis

At the end of the experiment, rats were fasted for 24 h with free access to water then sacrificed by cervical dislocation, the abdomen was opened, and the stomach was identified. Then the esophageal end was tied, and the stomach was excised and cut along the greater curvature. Gastric juice was collected and centrifuged at 3000 rpm for 10 min and the supernatant was used for estimation of the volume of gastric juice and the titratable acidity (Khushtar et al. 2009). Finally, the stomach was washed by warm saline and divided into two halves. The first half was placed in 10% neutral formalin for histological and immunohistochemical examination. The second half was sliced and kept in phosphate-buffered solution (PBS) and homogenized using a Teflon homogenizer (Polytron, Heidolph RZR 1, Germany). The mixture then centrifuged at 4500 rpm for 15 min at 4 °C (Tayeby et al. 2017). The supernatant was divided into aliquots and stored at −80 °C. Subsequently, the supernatant was used for the estimation of prostaglandin E2 (PGE2), malondialdehyde (MDA), reduced glutathione (GSH), tumor necrosis factor (TNF-α), interleukin 6 (IL6) and interleukin 10 (IL10) concentrations, and superoxide dismutase (SOD) activity.

### Measurement of total acid content (titratable acidity)

The total acid content of the gastric juice was measured by titrating it with 0.01 N NaOH using end point of pH 7.0 and was expressed as mEq/l (Khushtar et al. 2009).

### Measurement of the levels of PGE2 and TNF-α, IL-6, and IL-10

Prostaglandin E2 concentration was measured in the stomach homogenate by using the PGE2 enzyme-linked immunosorbent assay (ELISA) Kit (DRG International, Inc., USA) according to Wang et al. (2011). TNF-α (Chauvelot-Moachon et al. 1996), IL-6, and IL-10 were estimated by ELISA kit (Assaypro LLC, Charles, MO, USA for TNF-α and Sigma Chemical Company, USA for IL-6 and IL-10) in the stomach homogenate according to manufacturer instructions

### Determination of malondialdehyde (MDA), reduced glutathione levels (GSH), and superoxide dismutase (SOD) activity in gastric homogenate

Commercial kit (Biodiagnostic Company, Egypt) was used for estimation of MDA by trichloroacetic acid reaction (Buege and Aust 1978). Superoxide dismutase activity in gastric homogenate was determined according to Misra and Fridovich (1972) by inhibition of autoxidation of adrenaline at pH 10.2 at 30 °C. The levels of GSH in gastric homogenate were determined by using Ellman’s reaction using 5′5′-dithio-bis-2-nitrobenzoic acid (Biodiagnostic Company, Egypt). The absorbance was measured by using spectrophotometry (Shimadzu, UV-PC160, Japan) and wavelength 412 nm (Faure and Lafond 1995).

### Histological and immunohistochemical investigation

The samples were fixed in 10% neutral-buffered formalin for routine histological paraffin embedding. Sections of 5-μm thickness were stained with the routine hematoxylin and eosin (H&E) (Bancroft and Gamble 2008), and examined by using the light microscope.

### Immunohistochemical staining nuclear factor kappa, caspase-3, and proliferating cell nuclear antigen

For immunohistochemical studies, formalin-fixed paraffin-embedded tissue sections were deparaffinized, endogenous peroxidase activity was blocked with H_2_O_2_ in methanol, and the sections were heated in 0.01 mol/l citrate buffer in a microwave pressure cooker for 20 min. The slides were allowed to cool to room temperature, and nonspecific binding was blocked with normal horse serum for 20 min at room temperature. Then the slides were incubated with MIB-1 monoclonal antibody that was used for detection of caspase-3 (Cat #MA1-16843, Lot #QG2055501; Thermo Fisher, Fremont, CA, USA) at a dilution rate of 1:500, and polyclonal antibody p65 (Thermo Fisher Scientific, Catalog number RB-3034-PO) for detection of the nuclear factor kappa-light chain enhancer of the activated B cell (NF-κB) (Bancroft and Gamble 2008). Then the slides were washed with TBS and incubated with the corresponding secondary antibody, which were washed with TBS and maintained in 0.02% diaminobenzidine (DAB) with 0.01% H_2_O_2_ for 10 min. Counterstaining was performed with Mayer’s hematoxylin (Cat. #94585; BioGenex, Menarini Diagnostics, Antony, France) (Suvarna et al. 2018).

Proliferating cell nuclear antigen (PCNA) was carried out by using avidin-biotin peroxidase technique for localization of proliferating cell nuclear antigen by using primary antibodies (anti-PCNA monoclonal antibody dilution at a dilution rate 1:400, obtained from Lab Vision Company). Slides were counterstained with hematoxylin before mounting. The negative control sections were prepared by excluding the primary antibodies (Sharma and Gandhi 2012). The percentage of the positively stained cells was calculated by using light microscope at ×40 magnification.

### Ulcer score and ulcer index

The total ulcer surface area was measured from the photographs after considering the drawing scale. Ulcer severity was scored by the sum of the total ulcer surface area in the glandular portion of the stomach. The gastric lesions were scored between 0 and 5 according to their severity: 0 means no damage, 1 means blood at the lumen, 2 means pin-pointed erosions, 3 means 1–5 small erosions, 4 means several large erosions, and 5 means several large erosions with stomach perforation. The calculation of the ulcer score was performed according to Palle et al. (2018). The index was calculated by multiplying the average number of ulcers per stomach by the ulcer severity score and the percentage of animals with ulcers (ulcer index (UI) = the mean ulcer score of similarly treated animals × % of ulcerated animals of the group.).

### Statistical analysis

The data were expressed as means ± standard error of means (SEM). The statistical analysis was carried out using SPSS program (Chicago, USA, SPSS Inc.) version 16. Our results were analyzed using one‑way ANOVA (analysis of variance) followed by the post hoc Tukey’s multiple comparison tests for determination of the significance of the difference. It was considered significant at *P* value <0.05.

## Results

Our work indicated that BPA has a toxic effect on different gastric functions, and it elevated the levels of oxidant MDA and many inflammatory mediators like TNF-α and IL-6 with concomitant decrease in the anti-oxidant enzyme SOD and the anti-inflammatory markers IL10 and GSH. On the other hand, the natural plant MOLE has a protective effect to different gastric functions, as it improved the oxidative stress induced by BPA in the form of elevation of SOD and GSH with decrease in the MDA. It also increased the anti-inflammatory markers IL10 and GSH and decreased the inflammatory marker IL6. Also, our data revealed that persons taking MOLE as a routine intake will be protected more than persons taking it as a medication.

**Fig. 2:**
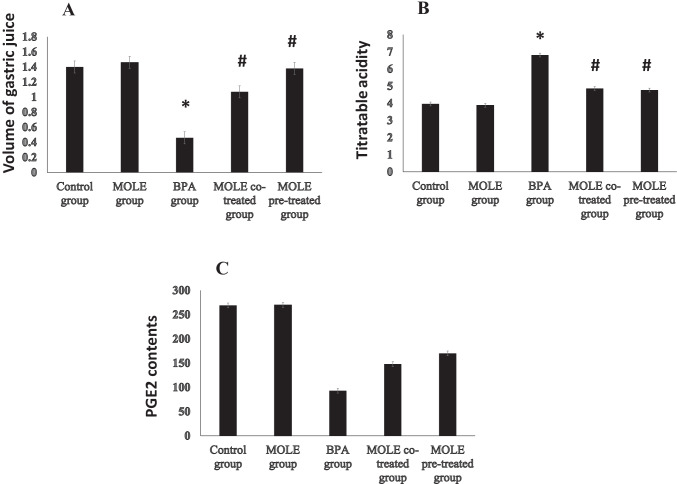
The effect of BPA and /or MOLE on the volume of gastric juice (**A**), titratable acidity (**B**), and PGE2 contents in the gastric tissues (**C**). Data are expressed as mean ± SEM (*n* = 8). ^*^Significant vs control group, ^#^significant vs BPA group, ^$^significant vs MOLE co-treated group

Figure 2 shows the effects of BPA and/or MOLE on the gastric functions. BPA significantly (*P* < 0.001) decreased the volume of gastric juice (Fig. 2A) and PGE2 (Fig. 2C) contents in the gastric tissue while it significantly (*P* < 0.001) increased the titratable acidity compared with the control group (Fig. 2B). On the contrast, administration of MOLE to BPA-intoxicated rats either before (pretreatment) or simultaneously (co-treatment) significantly (*P* < 0.001) increased the volume of the gastric juice (Fig. 2A) and PGE2 contents (Fig. 2C) in the gastric tissue and significantly (*P* < 0.001) decreased the titratable acidity (Fig. 2B) compared with the BPA-intoxicated group. In addition, pretreatment of BPA-intoxicated rats with MOLE (MOLE pre-treated group) significantly (*P* < 0.01) increased PGE2 contents in the gastric tissues compared to rats with MOLE co-treated group simultaneously.

**Fig. 3:**
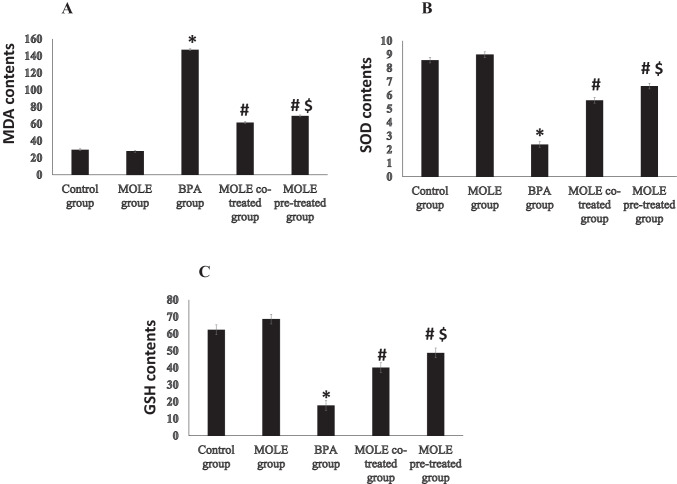
The effect of BPA and /or MOLE on the MDA (**A**), SOD (**B**), and GSH contents (**C**), on the gastric homogenate of bisphenol-treated rats. Data are expressed as mean ± SEM (*n* = 8). ^*^Significant vs control group, ^#^significant vs BPA group, ^$^significant vs MOLE co-treated group

Figure 3 presents the effects of BPA and/or MOLE on MDA and GSH contents and SOD activity in the gastric tissues. BPA significantly (*P* < 0.001) increased the MDA (Fig. 3A) content; however, it significantly (*P* < 0.001) decreased SOD activity (Fig. 3B) and GSH (Fig. 3C) content in the gastric tissues compared to the control group. On contrast, administration of MOLE to BPA-intoxicated rats either before or simultaneously significantly (*P* < 0.001) decreased the MDA content MDA (Fig. 3A) while it significantly (*P* < 0.001) increased SOD (Fig. 3B) and GSH content (Fig. 3C) activity in the gastric tissues compared to BPA group. In addition, the pretreatment of BPA-intoxicated rats with MOLE significantly (*P* < 0.01) decreased MDA content while it significantly (*P* < 0.01) increased GSH content and SOD activity compared to MOLE co-treated group.

**Fig. 4:**
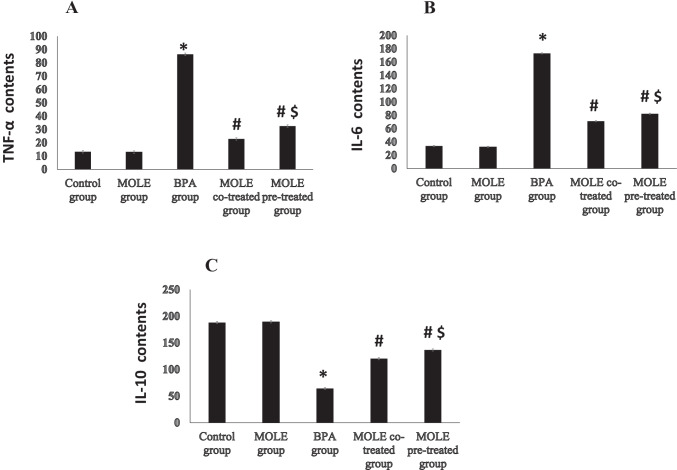
The effect of BPA and/or MOLE on the TNF-α (**A**), IL-6 (**B**), and IL-10 (**C**), contents in the gastric homogenate. Data are expressed as mean ± SEM (*n* = 8). ^*^Significant vs control group, ^#^significant vs BPA group, ^$^significant vs MOLE co-treated group

Figure 4 illustrates the effects of BPA and/or MOLE on TNF-α, IL-6, and IL-10 concentrations in the gastric tissues. BPA significantly (*P* < 0.001) increased the contents of both TNF-α and IL-6 (Fig. 4A and B) while it significantly (*P* < 0.001) decreased IL-10 (Fig. 4C) in the gastric tissues compared to the control group. On the contrast, administration of MOLE to BPA-intoxicated rats either before or with BPA significantly (*P* < 0.001) decreased the TNF-α and IL-6 contents (Fig. 4A and B) while significantly (*P* < 0.001) increased IL-10 content (Fig. 4C) in the gastric tissues compared with BPA-intoxicated group. In addition, the pretreatment of BPA-administrated rats with MOLE in MOLE pre-treated group significantly (*P* < 0.001) decreased TNF-α and IL-6 contents while it significantly (*P* < 0.001) increased IL-10 content in the gastric tissues when compared to MOLE co-treated group.

**Fig. 5:**
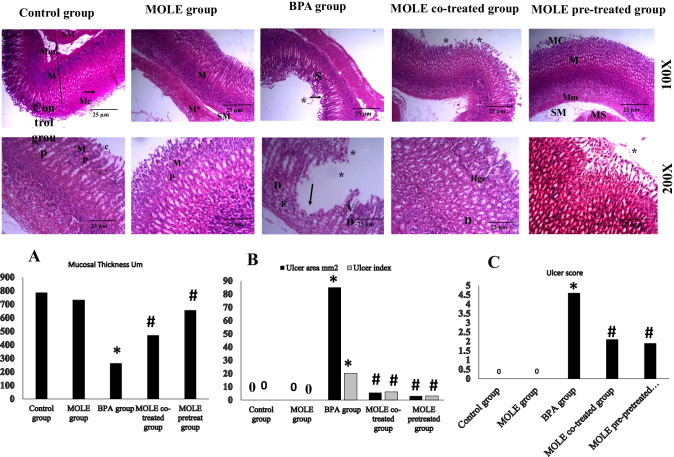
The H&E (100 × and 200 ×) staining of the gastric wall; in control and MOLE groups there is normal mucosa (M), muscularis mucosa (mm), submucosa (SM), and musculosa (M*). The mucosa has long, straight, packed gastric glands lined by normal surface columnar cells with basal oval nuclei (C). Also, mucous neck cells with basal flattened nuclei and parietal cells with central rounded nuclei and short narrow gastric pits (arrow) with intact mucous coat (MC). The BPA group shows a wide area of epithelial discontinuity (ulcer, arrow); exfoliated cells are seen within the gastric lumen (star) with wide separation of the fundic glands (S) and hemorrhage between them. Dark-stained (pychnosis) nuclei of some cells (P) with vacuolation of the cytoplasm other cells can be seen. The MOLE co-treated group shows slightly normal fundic glands with hemorrhage (hge) in between them and exfoliated cells in the lumen (star). The MOLE pre-treated group shows intact mucosa, submucosa, and musculosa; intact mucous coat (MC); and fundic glands but exfoliate cells into the lumen (star) and inflammatory cells in between the glands also can be seen (star). Panel **A** shows mucosal thickness, panel **B** shows the size of ulcerated area in mm^2^ and ulcer index, and panel **C** shows ulcer score in different groups. Data are expressed as mean ± SEM (*n* = 8). ^*^Significant vs control group, ^#^significant vs BPA group, ^$^significant vs MOLE co-treated group

Figure 5 shows the effects of BPA and/or MOLE on the microscopic structure of the stomach wall (H&E 100× and 200× stain). The mucosa, submucosa, and musculosa of the stomach of the control and MOLE-treated groups appear normal with long straight packed gastric glands. The stomach wall of BPA group showed wide area of epithelial discontinuity (ulceration) exfoliated cells within gastric lumen and wide separated gastric glands with hemorrhage between them. Also, dark-stained nuclei of some cells with vacuolation of the cytoplasm were observed in other cells. The stomach of MOLE co-treated group showed normal fundic glands with exfoliated cells into the lumen; there is dilated fundic gland with hemorrhage in between them. Whereas the stomach wall of MOLE pre-treated group showed intact mucosa, submucosa, and musculosa, with intact fundic glands but exfoliated cells in the lumen and inflammatory cells between the glands. In the same figure, panel A shows mucosal thickness in different groups. It shows significant decrease in the thickness of the mucosa in BPA group but treatment with MOLE increased the thickness with significant improvement in MOLE pre-treated group. Also, panels B and C show the ulcer area, ulcer index, and ulcer score in all groups; as seen there is no ulcer in the control and MOLE groups, but BPA group showed significant ulcer area and ulcer score. Treatment with MOLE decreased the ulcer area and ulcer score significantly when compared to BPA group.

**Fig. 6:**
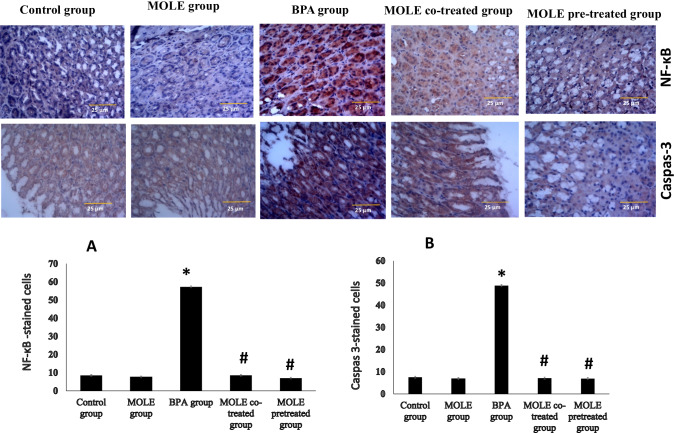
The immunohistochemical staining of the fundic glands with NF-κB (× 200) and caspase-3 (× 200) stains. In control and MOLE groups the figure shows very weak cytoplasmic immunoreactivity for NF‑κB in the cell lining of gastric glands. In BPA group there is a STRONG cytoplasmic immunoreactivity while in MOLE co-treated group the figure shows moderate cytoplasmic immunoreactivity but in MOLE pretreated group it shows WEAK cytoplasmic immunoreactivity for NF‑κB in the cell lining gastric glands. The figure also shows caspase-3 staining of a longitudinal section, the fundic glands of the stomach; in control and MOLE groups, it shows weak cytoplasmic immunoreactivity in the cell lining of gastric glands. In BPA group there is a STRONG cytoplasmic immunoreactivity; in MOLE co-treated group it appears with moderate cytoplasmic immunoreactivity but in MOLE pre-treated group it shows weak cytoplasmic immunoreactivity for caspase-3 in the cell lining of gastric glands. Panels **A** and **B** illustrate the same findings. Data are expressed as mean ± SEM (*n* = 8). ^*^Significant vs control group, ^#^significant vs BPA group, ^$^significant vs MOLE co-treated group

Figure 6 shows protein expression of NF-κB and caspase-3 in the fundic glands by immunohistochemical study. The photomicrograph of a transverse section of fundic glands of the stomach of the control and MOLE-treated groups showed very weak cytoplasmic immunoreactivity for NF‑κB and caspase-3 in the cell lining of gastric glands. While the photomicrograph of a transverse section of fundic glands of the stomach of BPA group showed strong cytoplasmic immunoreactivity for them. The MOLE co-treated group showed moderate cytoplasmic immunoreactivity for both NF‑κB and caspase-3, whereas that of the MOLE pre-treated group showed weak cytoplasmic immunoreactivity for NF‑κB and caspase-3. The same was expressed in the figure panels A and B.

**Fig. 7:**
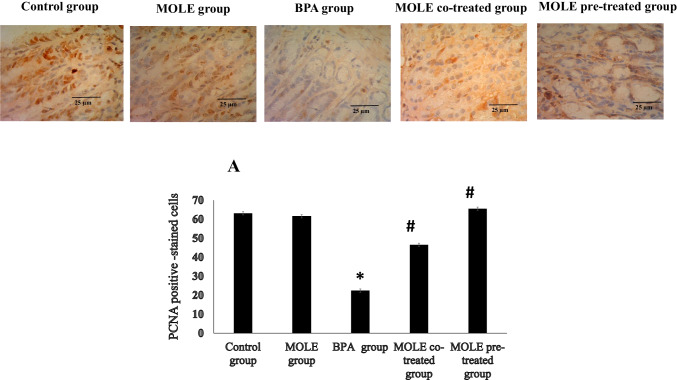
The immunohistochemical staining with PCNA with counterstain hematoxylin (× 400) stain. In the fundic gastric mucosa control and MOLE groups show very strong positive brown PCNA immunoreactivity in many nuclei of cell lining. In BPA group there is a very weak immunoreactivity in few nuclei of cell lining. In MOLE co-treated group the figure shows moderate immunoreactivity but in MOLE pre-treated group it shows very strong immunoreactivity. Data are expressed as mean ± SEM (*n* = 8). ^*^Significant vs control group, ^#^significant vs BPA group, ^$^significant vs MOLE co-treated group

Figure 7 shows the immunoreactivity for PCNA protein expression. The fundic gastric mucosa of the control and MOLE-treated groups showed very strong immunoreactivity for PCNA in many nuclei of cell lining of gastric mucosa. While that of BPA-intoxicated rats showed very weak immunoreactivity in few nuclei of cell lining of the gastric mucosa. The fundic gastric mucosa of rats administrated MOLE and BPA simultaneously in MOLE co-treated group; there is moderate immunoreactivity for PCNA. The fundic gastric mucosa of MOLE pre-treated group showed very strong immunoreactivity for PCNA. Panel A illustrates the immunoreactivity to PCNA in different groups.

## Discussion

Gastric ulcer is one of the most predominant gastrointestinal diseases affecting humans worldwide. Special interest must be given to find natural sources to protect and treat gastric ulcers. Plant and herb natural extracts are more preferred because of their wide safety margins and less or no adverse health effects (Alhakmani et al. 2013). Wide spreading use of plastic products expose human being to large amounts of toxic products like BPA, which was approved to disturb endocrine as well as the nervous systems (Szymanska et al. 2018; Makowska and Gonkowski 2020). The gastric effects of BPA were not sufficiently studied. Also, the use of known anti-ulcer drugs as proton pump inhibitors have shown many side effects, so the use of natural drugs will be good alternative with minimal side effect. For many years, traditional healers had used different parts of *Moringa oleifera* as a nutrient dense food source, in addition encourage its use for treatment of many diseases.

Our data revealed that BPA decreased gastric mucosal thickness, the volume of gastric juice, and prostaglandin content in the gastric tissue. However, it increased ulcer area, ulcer index, ulcer score, and titratable acidity (acid secretion), which subsequently resulted in desquamation of the gastric epithelium with ulceration and hemorrhage between damaged gastric glands; these results agreed with Makowska and Gonkowski (2020) and Necheles et al. (1938), who reported that toxic effects of BPA may attribute to the neurotoxic and neurodegenerative effects of BPA on the enteric nervous system (ENS) as it has been indicated that BPA harms DNA and consequently altered gene expression of mediators in the nervous system including the ENS. Also, BPA decreases the number of the cholinergic neurons decreasing the acetylcholine and subsequently decreases the gastric secretion. In addition, Szymanska et al. (2018) stated that BPA could induce smooth muscle relaxation with a decrease in its neuroprotective effect. The ulcerative effect of BPA can be attributed to the induction of the oxidative stress that is indicated by an increase in the gastric MDA levels with decrease in gastric GSH content and SOD activity. These findings were in line with that of Ozaydın et al. (2018), who found that oral administration of BPA to rats with different doses for 8 weeks decreases plasma levels of GSH, SOD, GPx, and CAT. Free radicals play roles in toxic chemical-induced cellular damage that results in cellular injury and damage of gastric mucosal anti-oxidants and consequently damage and ulceration of gastric mucosa (Gassman 2017). However, GSH protects the cells from BPA-induced oxidative by direct removal of the oxidants or by enzymes as glutathione peroxidase, leading to consumption of glutathione and its conversion into ineffective form of disulfate (Sabour 2019). The ulcerative effect of BPA can be explained by the activation of the inflammatory response that was indicated by an increase in the TNF-α and IL-6 with a decrease in IL-10 contents in the gastric tissue. BPA is known as endocrine disruptor mimicking estrogenic activity; it can induce immune dysregulation by affecting the immune cell signaling pathways and the immune responses. In support with these finding, Kharrazian (2014) had reported that BPA promotes signaling of T cell in autoimmune diseases and can stimulate the macrophage production of TNF-α. Increased production of TNF-α was linked to gastric ulcer and cancer as it contributes to mucosal injury (Sugimoto et al. 2007). Moreover, TNF-α activates caspase-3 that leads to gastric cell apoptosis (Park et al. 2000) and ulcer formation; this was in agreement with Zhang et al. (2020), who mentioned that BPA can also increase the expression of IL-6 by upregulation of NF-κB (Zhang et al. 2020). Moreover, it has an inhibitory activity on T-helper type-2 cell-related cytokine IL-10 (Lee et al. 2010).

On the contrast, administration of rats with MOLE before or simultaneously with BPA exerted gastric cytoprotective effect as it ameliorated the toxic effects of BPA on the gastric mucosal thickness, the volume of gastric juice, prostaglandin contents, the ulcer area, ulcer index, and the titratable acidity (acid secretion), as well as it protects the gastric glands with intact mucosa, which was more prominent in rats pretreated with MOLE. These findings were in accordance with those of the previous study, which revealed anti-ulcer effect of *Moringa* against indomethacin-induced gastric ulcer (Almuzafar 2018). These protective effects of MOLE against BPA-induced toxic effects on the stomach may be related to increased prostaglandin secretion as it is an important factor in ulcer prevention because it increases the local blood flow and increases both mucus and bicarbonate secretion, thus protecting mucosal integrity (Cryer and Mahaffey 2014). Furthermore, MOLE has powerful anti-oxidant activities as the current study showed that treatment of rats with MOLE decreased gastric content of MDA while it increased SOD activity and GSH content in the gastric homogenate. These findings were in line with previous studies, which indicated that *Moringa* decreases lipid peroxidation biomarker, MDA, while increases the activities of the anti-oxidant enzymes (GPX, CAT, SOD) (Mbikay 2012). Also, Owoade et al. (2017) and Abd Eldaim et al. (2017) indicated that *Moringa oleifera* regenerates hepatic and renal SOD, CAT, and GSH activities in diabetic rats due to decreased production of reactive oxygen species. The anti-oxidant effects of MOLE may be attributed to its contents of phytoconstituents such as polyphenols; flavonoids including quercetin, ellagic acid, kaempferol, and apigenin; and tannins, which scavenge free radicals, inhibit oxidases, and activate the anti-oxidant enzymes (Luqman et al. 2012). Also, it either increase the biosynthesis of anti-oxidants like GSH or reduce the extent of oxidative stress that leads to less cellular degradation (Fotio et al. 2020).

Moreover, the results of the current study revealed that treatment of BPA-intoxicated rats with MOLE had anti-inflammatory effects as it decreased TNF-α, IL-6, and NF-κB while increased IL-10 levels in gastric homogenate; this was in agreement with Muangnoi et al. (2012) and Tan et al. (2015), who reported that the inhibitory effect of MOLE on BPA-induced production of TNF-α may be due to its enhancement effect of the production of prostaglandin, the potent inhibitor of TNF-α release also; it inhibits NF-κB activation by blocking the degradation of IκB-α. Thus, it prevents the NF-κB in the cytoplasm from further activation. Pretreatment of BPA-intoxicated rats with MOLE had potent anti-inflammatory than co-treatment of rats with both BPA and MOLE.

Cell proliferation plays an important role in the healing of the gastric ulcers. Mucosal cell regeneration occurs by proliferation of undifferentiated epithelial cells, which migrate to cover the base of the ulcer. The balance between proliferation and apoptosis is important for the maintenance of epithelium. Without a continuous epithelial barrier, the mucosa would be exposed to infection and chemical injury leading to prevention of the ulcer healing (Negroni et al. 2015). PCNA is a nuclear peptide that is expressed during cell proliferation (Vasconcelos et al. 2010). The present results revealed that BPA decreased the number of the mucosal PCNA positive cells with increase of caspase-3, while MOLE-treated group increased PCNA positive cells with decrease of caspase-3. In line with these findings, Sinha et al. (2012) reported that MOLE could produce its anti-ulcer effect not only by inducing intensified mucosal proliferation but also by inhibiting apoptosis.

## Conclusion

Bisphenol A induced gastric ulceration through decreasing the production of prostaglandin E2 (PGE2) and the proliferation of gastric mucosal cells and inducing oxidative stress and protein expression of pro-inflammatory cytokines and pro-apoptotic biomarker in the gastric tissue. However MOLE prevented gastric ulceration via increasing the production of PGE2, gastric mucosal cell proliferation, and its anti-oxidant and anti-inflammatory activities.

## Data Availability

All data used in this study are included in this published article.
